# Enhancement of Visual Motion Detection Thresholds in Early Deaf People

**DOI:** 10.1371/journal.pone.0090498

**Published:** 2014-02-28

**Authors:** Martha M. Shiell, François Champoux, Robert J. Zatorre

**Affiliations:** 1 Montreal Neurological Institute, McGill University, Montreal, Quebec, Canada; 2 BRAMS: International Laboratory for Brain, Music, and Sound Research, Montreal, Quebec, Canada; 3 CRBLM Centre for Research on Brain, Language, and Music, Montreal, Quebec, Canada; 4 École d’orthophonie et d’audiologie, Université de Montréal, Montreal, Quebec, Canada; 5 Centre de recherche interdisciplinaire en réadaptation du Montréal métropolitain – Institut Raymond Dewar, Montreal, Quebec, Canada; University of Montreal, Canada

## Abstract

In deaf people, the auditory cortex can reorganize to support visual motion processing. Although this cross-modal reorganization has long been thought to subserve enhanced visual abilities, previous research has been unsuccessful at identifying behavioural enhancements specific to motion processing. Recently, research with congenitally deaf cats has uncovered an enhancement for visual motion detection. Our goal was to test for a similar difference between deaf and hearing people. We tested 16 early and profoundly deaf participants and 20 hearing controls. Participants completed a visual motion detection task, in which they were asked to determine which of two sinusoidal gratings was moving. The speed of the moving grating varied according to an adaptive staircase procedure, allowing us to determine the lowest speed necessary for participants to detect motion. Consistent with previous research in deaf cats, the deaf group had lower motion detection thresholds than the hearing. This finding supports the proposal that cross-modal reorganization after sensory deprivation will occur for supramodal sensory features and preserve the output functions.

## Introduction

Most people are familiar with the popular idea that the loss of one sense, such as vision or audition, can lead to enhancements of the remaining senses. This idea is supported anecdotally and scientifically, particularly in the domain of visual deprivation. In blind people, researchers have documented behavioural enhancements to specific aspects of auditory, tactile, and olfactory processing (for reviews, see [1], [2]). In some instances, these enhancements have been attributed directly to cross-modal processing in the visual cortex – with no visual input, brain regions that are normally devoted to vision reorganize to process incoming auditory information (e.g. [3], [4]). Similar cross-modal neural recruitment has been found in deaf people, with increased responsiveness of the superior temporal cortex in response to visual information, specifically to visual motion stimuli [5]–[10]. Although this cross-modal activity in deaf people is believed to support some enhanced visual abilities [11], the exact behavioural correlates of this activity have yet to be fully understood.

One barrier to understanding the nature of cross-modal activity in deaf people has been the difficulty in identifying specific behavioural enhancements in this population. Many studies have identified changes to visual attention (for a review, see [12]), but researchers have been less successful at confirming behavioural enhancements that are specific to visual motion processing. For example, deaf people show no enhancement for detecting changes in velocity [13], and the evidence for enhancements to motion direction sensitivity is ambiguous: while one recent study found that deaf people were faster and more accurate at discriminating small differences in the angle of motion direction in the periphery [14], other reports found that coherent motion direction thresholds were either similar to or worse than in hearing people [15], [16]. Although deaf people sometimes show faster reaction times to moving stimuli in the periphery as compared to hearing people [17]–[19] (but see [14] for a counter example), they also show faster reaction times for stationary peripheral stimuli [21]–[23] suggesting the possibility of a general enhancement to reaction times for peripheral stimuli rather than a specific change to visual motion processing. Consistent with this hypothesis, deaf people show a bias for peripheral space in the distribution of visual attention [24], [25], and in the distribution of ganglion cells in the retina [26].

Recently, the link between cross-modal cortical recruitment and visual ability has been clarified in congenitally deaf cats [27]. Here, researchers found that auditory deprivation led to an enhancement in two aspects of vision: motion detection and peripheral localization. Consistent with previous work in deaf humans, there were no changes in the cat’s sensitivity to motion direction or velocity, nor to non-motion related features, such as grating acuity, vernier acuity, and orientation. These selective visual enhancements were abolished when auditory regions were deactivated, confirming that they were supported by cross-modal neural activity [27].

In the present study, we sought to replicate the behavioural enhancement in visual motion detection previously found in deaf cats [27] in a group of deaf humans. Despite the abundance of studies examining visual motion processing in deaf people, to our knowledge, none have specifically measured motion detection thresholds, the variable known to be enhanced in deaf cats. This link between non-human and human research is a necessary step towards understanding cross-modal plasticity in humans. This issue is particularly relevant to further our understanding of plasticity in the auditory system, where humans show unique cognitive abilities for auditory information, such as language and music processing. We hypothesized that, similarly to deaf cats, deaf humans will show enhanced visual motion detection thresholds when compared to hearing controls when measured with similar psychophysical approaches.

## Materials and Methods

### Ethics Statement

The experiment was approved by the Research Ethics Board at the Montreal Neurological Institute and all participants gave informed written consent.

### Participants

Twenty hearing (13 males, 7 females; mean age: 31.8 years old) and 16 deaf people (9 male, 7 female; mean age: 31.2 years old) participated in the study. All participants had normal or corrected-to-normal vision and no history of neurological conditions. From the deaf group, seven participants reported suspected hereditary congenital deafness, seven reported congenital deafness of unknown etiology, and two were deafened from meningitis at 11 and 6 months of age. All deaf participants were confirmed to have profound hearing loss using standard audiometry [28]: Pure-tone thresholds at all frequencies tested (500, 1000, 2000, 4000 and 8000 Hz) were above 90 dB for each participant, with the exception of four participants who were able to hear 500 Hz at 80 or 85 dB. Deaf participants used sign language as their primary language and had minimal hearing-aid use. Hearing participants reported normal hearing and no experience with sign language or lipreading.

### Stimuli

**Figure 1:**
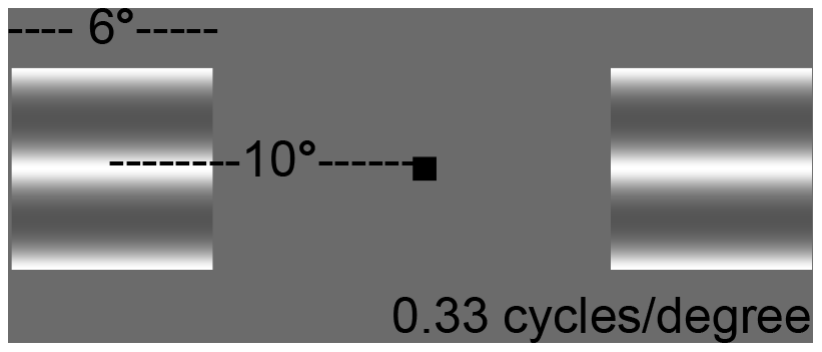
Diagram of the visual motion detection task. In each trial, two gratings appeared in the left and right visual fields centred at an eccentricity of ten degrees for 500 ms. One of the gratings was stationary while the other moved. Participants indicated which of the two gratings was moving by a button press. The speed of the moving grating varied according to an adaptive staircase procedure.

Stimuli were two greyscale, horizontally-oriented sinusoidal gratings (grating size: 6°×6°, spatial frequency: 0.33 cycle/°, Michelson contrast: 50%), presented on a CRT computer monitory with a grey background. A schematic of the stimulus is shown in figure 1. Gratings were presented simultaneously for 500 ms in the left and right visuals fields, centred at −10° and +10° from a central fixation square (size: 0.5°×0.5°). In each trial, one the two gratings was randomly selected to move while the other remained stationary. The motion of the moving grating was randomized between upwards and downwards. The speed of the motion varied according to an adaptive staircase procedure, described below. The stimuli and staircase procedure were generated using Presentation software (Neurobehavioral Systems, Albany, CA, USA).

### Procedure

Participants were tested individually. The experimenter verbally explained and demonstrated the task, with the assistance of a sign language interpreter when necessary. Participants were instructed to maintain fixation on the central square and indicate, by button-press, which of the two gratings (the left or the right) was moving, and to guess if uncertain. Participants were then seated alone in a darkened room, with their heads stabilized in a chin rest, positioned 57 cm from the centre of the computer screen. We used an Eyelink 1000 eye tracker (SR Research, Mississauga, ON, Canada) with a 1000 Hz sampling rate to monitor monocular eye movement of the right eye during the task. The eye tracker was calibrated before each run. The first run consisted of a practice, where participants were given automatic feedback on their responses, as well as feedback from the experimenter on their fixation. The participants then completed eight testing runs, broken up with two breaks during which the experimenter interviewed the participant on their history and did audiometric testing for hearing thresholds.

For the first trial of each run, the grating had a speed of 0.566 degrees/second. For each correct response, the speed decreased by 0.0472 degrees/second, and for each incorrect response, the speed increased by 0.142 degrees/second. Thus the staircase consisted of a one-up one-down procedure, weighted with a 1∶3 ratio in step size [29]. Trials in which participants broke fixation during the presentation of the gratings were automatically excluded from the staircase. If the staircase reached a speed of zero or 0.660 degrees/second, the subsequent trial was automatically one step up or down, respectively. The run terminated after 15 reversals, which took an average of 136.7 trials (range: 94–211, standard deviation: 19.2). For each participant, individual runs were discarded if the participant broke fixation in more than 18% of the trials. This cut-off was chosen because it represents two standard deviations above the mean number of times fixation was broken across all participants and runs. Fourteen runs were excluded with this criterion: seven participants (3 hearing, 4 deaf) had one run excluded, two had two excluded (1 deaf, 1 hearing), and 1 (deaf) had three excluded. Within a run, the output threshold consisted of the arithmetic mean of all 15 reversals. Across a participant’s set of runs, we took the median value as that participant’s motion detection threshold.

## Results

**Figure 2:**
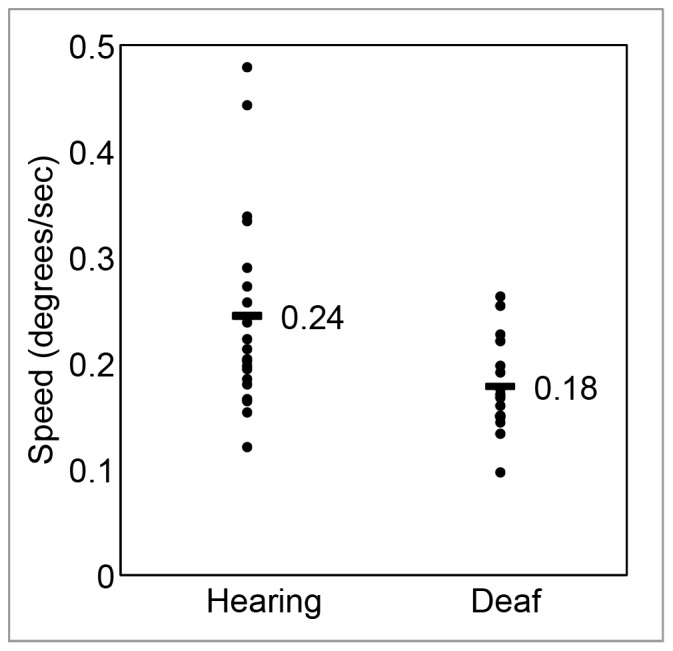
Visual motion detection thresholds of the hearing and deaf groups. Group averages are shown with horizontal bars. Deaf people showed significantly lower motion detection thresholds than hearing people. Effect remained statistically significant when two possible outliers in the upper range of the hearing group were excluded.

To compare motion detection thresholds of deaf and hearing groups, we did a one-tailed Student’s *t*-test. As shown in figure 2, the deaf group had lower visual motion detection thresholds than the hearing group (*t* = 2.71, *p* = 0.0055). Visual inspection of the data showed two hearing participants with particularly high thresholds. Exclusion of these two individuals did not eliminate the statistical difference between groups (*t* = 2.19, *p* = 0.018 DF = 32). A Shapiro-Wilks test indicated that the hearing group was not normally distributed when all participants were included (*W* = 0.813, *p* = 0.013). As such, we repeated the group comparison with the non-parametric Mann-Whitney test, and the statistical significance of the group difference was preserved (*U* = 82.5, *p* = 0.007 with all participants included; *U* = 82.5, *p* = 0.017 with two suspected outliers in the hearing group excluded).

## Discussion

We found that people with early and profound deafness have lower thresholds for visual motion detection compared to people with normal hearing. This finding is consistent with previous research in congenitally deaf cats, where this enhanced visual ability is supported by activity in auditory regions [27]. Based on this previous research [27], we believe that the enhanced visual ability found in deaf humans is likewise supported by auditory-visual cross-modal neural activity. Our proposal is corroborated by the well-documented cross-modal recruitment of auditory regions for visual motion processing in deaf people [5]–[10]. It is also consistent with research in blind people, where selective enhancements in performance on auditory behavioural tasks have been linked to cross-modal activity in regions that normally process vision (for a review, see [30]). However, given that we tested behaviour without measuring neural activity, we cannot rule out other possible mechanisms that may play a part. In particular, documented changes to the distribution of retinal ganglion cells [26] and to early visual processing [22] may have contributed.

Our result contrasts with previous studies on aspects of visual motion processing in deaf people, where deaf and hearing performed similarly on tests of direction and velocity perception [13], [15], [16], [19], [20]. In fact, a review of all research on the visual abilities of deaf people reveals that enhancements are highly selective [11]. Several theories have been proposed to explain this selectivity. Early suggestions followed the idea that enhancements were limited according to the principles of organization in the visual system, restricted to functions of the dorsal visual stream [11], [31] or to the magnocellular system [17]. While either one of these suggestions is consistent with our result, both can be rejected in light of null results in other research. The dorsal stream hypothesis is not supported because it fails to explain the null results for visual motion direction and velocity processing [13], [15], [16], [19], [20], both of which occur in the dorsal visual stream [32]. The magnocellular hypothesis is not supported based on evidence that deaf and hearing people have similar contrast thresholds for the detection of motion [33], while the magnocellular system is known to support motion detection of gratings with low contrast [34]. Another early explanation for the specificity of visual enhancements in deaf people held that enhancements may be limited to attentionally-demanding tasks in peripheral vision [11]. While it is clear that there are changes to peripheral visual attention after deafness [12], we do not believe that these changes can explain our finding, as previous research that also used peripheral stimuli did not find a similar enhancement [13], [16], [19].

Based on the evidence from deaf cats, and other data, a more recent theory for the selectivity of visual enhancements in deaf people has been proposed [27], [35]. This idea, termed the supramodal hypothesis, can be explained in three parts. First, cross-modal neural recruitment after deafness will occur selectively in cortical modules that rely on supramodal input. “Supramodal input” refers to sensory features that are common to more than one sensory modality. For example, motion is a supramodal sensory feature because it can be sensed by both audition and vision, whereas color is not because it can be sensed only by vision [27]. Second, the output function of a cortical module will be preserved after cross-modal reorganization. This idea of preserved function is consistent with the proposal for a metamodal organization of the brain, determined based on output functions regardless of the input sensory modality [36]. A similar explanation of preserved function has been proposed in cross-modal research with blind people (e.g. [37], [38], for reviews see [30], [39]). Third, the reorganization will be limited to cortical modules for which audition is the primary sensory input [40]. This part of the hypothesis was introduced to explain the specificity of enhancements for motion processing, reasoning from the fact that both direction and velocity, two features for which no behavioural enhancements have been documented, are processed primarily in the visual system, with no known homologues in the auditory system [40].

Our result of enhanced visual motion detection in deaf people is consistent with the supramodal hypothesis, as motion can be perceived with both the visual and auditory senses. Provided that our behavioural result is supported by cross-modal recruitment of the auditory cortex, more research is needed to determine the exact function of the auditory cortical module that has been reorganized. According to the supramodal hypothesis, this hypothetical cortical module may be involved in peripheral auditory motion detection. Although the neural correlates specific to motion detection have not been examined in the auditory domain, previous research has examined the neural correlates of auditory motion processing. Within the temporal lobe, the planum temporale (PT) has been implicated in this function [41]–[43]. The PT also supports motion-related cross-modal activity in deaf people [5]–[8]. In a recent study from our lab [44], deaf people who had minimal hearing-aid use showed greater functional connectivity between the PT and primary visual cortex as compared to hearing people, which again suggests cross-modal reorganization of this region after auditory deprivation. The PT is therefore a fitting proposed neural correlate for the enhanced visual motion detection thresholds that we observed in the present study.

As our study examined the effects of early deafness, more research is needed to determine whether or not this sensory compensation will occur for deafness acquired later in life. As well, since our deaf and hearing groups differed in language experience, future research may explore whether or not visual spatial language experience impacts visual motion detection thresholds. Another intriguing question for future research concerns the interaction between visual ability and hearing rehabilitation: Since cross-modal reorganization of auditory cortex is known to interfere with the functioning of a cochlear-implant [45]–[48], we predict that cochlear-implant outcome and visual motion detection thresholds will correlate, such that individuals with worse cochlear-implant proficiency, presumably because of increased cross-modal reorganization, will also show better visual motion detection thresholds.
